# Supplementary material for: Experimental evidence that hyperthermia limits offspring provisioning in a temperate-breeding bird
Source: R Soc Open Sci. 2020 Oct 7;7(10):201589. doi: 10.1098/rsos.201589 (PMC7657879; doi:10.1098/rsos.201589)
Supplement: Feeding Rate Methods and Results - Online Supplementary Material [file rsos201589supp1.docx]

**Electronic Supplementary Material**

## Experimental evidence that hyperthermia limits offspring provisioning in a temperate breeding bird

^1^Environmental and Life Sciences Graduate Program, Trent University, 1600 West Bank Dr., Peterborough ON, Canada

^2^Faculty of Forestry and Environmental Management, University of New Brunswick, 28 Dineen Dr., Fredericton, NB, Canada

^3^Department of Biology, Trent University, 1600 West Bank Dr., Peterborough ON, Canada

**Calculating feeding rate**

To obtain the hourly feeding rate per chick (see statistical analysis below for explanation of why we chose per chick over total per hour), we first obtained the hourly feeding rate using the package feedr (LaZerte et al., 2017) and the function “visits” to transform raw radio-frequency identification (RFID) reads into visits to the nest box. We considered repeated reads from the same individual as a single visit if successive reads were < 60 s (i.e., threshold time) apart. While the threshold time has been shown to influence the estimate of the true number of feeding events (Vitousek et al., 2018), our chosen threshold time should not influence the interpretation of our results, because we assigned an equal number of birds to each treatment. We then calculated the hourly feeding rate per chick by summing the number of visits in each hour and dividing it by the brood size on the day of the respective feeding rate measurement. In our analysis, we only included hours in which the birds had at least one observation, to minimize any potential bias caused by human disturbance (e.g., during captures or nest checks), that may have resulted in birds being away from the box for at least one hour.

**Statistical analysis of feeding rate**

Initially we attempted to model feeding rate as visits·hr^-1^ with a Poisson error distribution, but the spread of the residuals significantly increased with predicted feeding rate, and we therefore opted to model feeding rate as visits·hr^-1^·chick^-1^ (i.e., visits·hr^-1^/ brood size; again, Gaussian distributed) with an applied combination of variance structures (see details below). This approach greatly improved the spread of our residuals.

Residual plots from the initial Poisson model suggested that variance in feeding rate differed with nestling age, as well as between treatments, and so we applied a combination of variance structures to the data to resolve the heteroskedasticity among nestling age and treatment. We compared the Akaike’s Information Criterion (AIC) scores of three different models with differing variance structures, as recommended by Zuur et al. (2009) to determine which variance structure provided the best fit. In model 1, we applied a fixed variance structure to nestling age. In model 2, we applied a constant variance structure to treatment, and in model 3, we applied a combination of the fixed and constant variance structures to nestling age and treatment respectively. Model 3 (the combined variance structure) had a substantially lower AIC score than the other two models (Model 1, AIC = 2722.675, Log *L*_ik_= 1350.337, *df* = 11; Model 2, AIC = 2704.275, Log *L*_ik_ = -1342.138, *df* = 10; Model 3, AIC = 2637.584, Log *L*_ik_= - 1307.792, *df* = 11).

The final model (with the combined variance structure) included fixed effects of treatment, hourly T_a_, nestling age, maternal age, hour (i.e., time of day), and an interaction term between treatment and hourly T_a_. We initially predicted feeding rate would vary quadratically across T_a_, so we ran and compared the log-likelihoods of two models, one in which T_a_ was assigned as a linear term and another in which T_a_ was assigned as a quadratic polynomial term. We found that the linear and quadratic models did not significantly differ from each other in their log-likelihoods (Log$\mathcal{L}$Linear T_a_: -1263.24, df = 11; Log$\mathcal{L}$ Quadratic T_a_ = -1262.49, df = 13, Likelihood Ratio = 1.51, *p* = 0.470), and so we opted to use the simpler model of a linear relationship between T_a_ and feeding rate. For all model comparisons, we estimated parameters with maximum likelihood, while for final models, we estimated parameters with restricted maximum likelihood (Zuur et al. 2009) In both the T_b_ and feeding rate models, variance explained by individual identification was estimated by inclusion of random intercepts, and a first order autoregressive structure was used to correct for non-independence between adjacent data points.

**Feeding Rate Results**

On average, trimmed and control birds did not differ in their feeding rates, mean ± 1 s.e.m. (i.e., Control: 1.94 ± 0.162; Trimmed: 2.36 ± 0.165; treatment, $\beta$ = -0.45, 95% CI [-1.45, 0.56], *t* = -0.96, *p* = 0.354). However, feeding rate differed between treatments with respect to T_a_ (i.e., Treatment x T_a_, $\beta$ = 0.04, 95% CI [0.00, 0.08], *t* = 2.02, *p* = 0.044, Figure S1): control birds decreased feeding rate with increasing T_a_ ($\beta$ = -0.03, 95% CI [-0.06, 0.00], *t* = 2.04, *p* = 0.042), while trimmed birds maintained a consistent feeding rate across T_a_ . Feeding rate increased with time of day (i.e., Hour, $\beta$ = 0.05, 95% CI [0.03, 0.08], *t* = 4.81, *p* < 0.001), and with nestling age ($\beta$ = 0.11, 95% CI [0.08, 0.14], *t* = 6.99, *p* < 0.001), but did not differ with maternal age ($\beta$ = 0.00, 95% CI [-0.51, -0.51], *t* = -0.01, *p* = 0.990).

**Figure S1** The effect of treatment (feather-trimming) on the relationship between feeding rate and ambient temperature in female tree swallows (*Tachycineta bicolor*), as determined via a linear mixed effects model. Trimmed birds had higher feeding rates than control birds at higher T_a_ (*p* = 0.044). Dots represent the average feeding rate per temperature (raw data), according to treatment.

**References**

LaZerte, S. E., Reudink, M. W., Otter, K. A., Kusack, J., Bailey, J. M., Woolverton, A., … Hill,

D. J. (2017). Feedr and animalnexus.ca: A paired R package and user-friendly Web application for transforming and visualizing animal movement data from static stations. *Ecology and Evolution*, *7*(19), 7884–7896. doi: [10.1002/ece3.3240](https://doi.org/10.1002/ece3.3240)

Vitousek, M. N., Taff, C. C., Ardia, D. R., Stedman, J. M., Zimmer, C., Salzman, T. C., &

Winkler, D. W. (2018). The lingering impact of stress: Brief acute glucocorticoid exposure has sustained, dose-dependent effects on reproduction. *Proceedings of the Royal Society B: Biological Sciences*, 285(1882), 20180722. doi: 10.1098/rspb.2018.0722

Zuur, A., Ieno, E. N., Walker, N., Saveiliev, A. A., & Smith, G. M. (2009). *Mixed effects models*

*and extensions in ecology with R*. New York: Springer.
